# OrMAC: A Hybrid MAC Protocol Using Orthogonal Codes for Channel Access in M2M Networks

**DOI:** 10.3390/s17092138

**Published:** 2017-09-17

**Authors:** Ethungshan Shitiri, In-Seop Park, Ho-Shin Cho

**Affiliations:** 1 School of Electronics Engineering, Kyungpook National University, Daegu 41556, Korea; ethungshan@ee.knu.ac.kr; 2 SK Hynix, Icheon 12813, Korea; autotale90@gmail.com

**Keywords:** hybrid, latency, medium access control, machine-to-machine, orthogonal code

## Abstract

This paper proposes a hybrid medium access protocol named **or**thogonal coded **m**edium **a**ccess **c**ontrol (OrMAC), which extends the principle of distributed queuing collision avoidance protocol (DQCA) of wireless local area network (WLAN) to delay-sensitive machine-to-machine (M2M) networks. OrMAC pre-assigns orthogonal codes, which serve as the channel contention signals, to the nodes entering the network. The “pre-assignment” eliminates contention collisions since it guarantees that no two nodes share the same contention code. Moreover, OrMAC employs a prioritized channel access by allowing nodes to control the transmission power of the contention signal depending on the delay sensitivity of the data. The power at which a contention signal arrives at the access point reflects the urgency of the packets waiting for transmission in the buffer. A contention signal with a high received power is assigned a high priority and vice versa for a contention signal with a low received power. Numerical experiments are carried out to compare the performance of OrMAC to that of DQCA in terms of the packet delivery ratio, latency, discarded packet ratio, and throughput. The results show that OrMAC can outperform DQCA in all the aforementioned performance metrics.

## 1. Introduction

Machine-to-machine (M2M) communication empowers devices to seamlessly exchange information with minimal or absolutely no human assistance and is highly regarded as the backbone of Internet of things [1]. Noteworthy applications of M2M networks from the small-scale personal domain to the intermediate-scale public domain and to the large-scale industrial domain are smart homes and smart healthcare, smart power grids and smart cities, and smart industrial automations, respectively [2,3,4].

From such a wide range of applications, one can easily discern the distinct characteristics of the networks envisioned for M2M communication: various traffic types; frequently varying traffic loads; large number of connected devices. The combination of these characteristics makes an M2M network ‘heterogeneous’ in every sense possible. For instance, both non-critical and time-critical traffic can coexist, or bursty and spontaneous traffics may flow simultaneously. Such heterogeneity poses a new set of networking complexities and challenges, particularly for sharing the channel resources in an ordered and efficient manner [3]. Therefore, given the high node density in an M2M network coupled with the heterogeneity, it is pertinent to design an efficient channel sharing technique. Conventional standalone medium access control (MAC) protocols, such as carrier sensing multiple access (CSMA) and time division multiple access (TDMA), cannot be directly applied to M2M networks since they cannot handle the aforementioned heterogeneity. For instance, CSMA suffers from high collision rates and relatively high overhead (e.g., CSMA/CA) during high traffic loads and TDMA suffers from low channel usage and low scalability during low traffic loads [4].

Combining the strengths of two standalone MAC protocols can mitigate their weaknesses, which has led to the study of hybrid MAC protocols [4,5,6,7]. This combination allows hybrid MAC protocols to seamlessly switch between two standalones, which forms the hybrid, when there is a change in the characteristic of the network. Therefore, hybrid MAC protocols tend to be robust to heterogeneity and this robustness makes them the front-runners of M2M MAC protocols. Over the years, several hybrid MAC protocols for M2M networks have been developed such as CSMA-TDMA hybrid [5], CSMA-TDMA hybrid with fairness provisioning [6], and CSMA/CA-PCF (point coordination function) hybrid [7]. CSMA-FDMA (frequency division multiple access) or CSMA-CDMA (code division multiple access) hybrids are feasible; however, these types of hybrid protocols can incur high hardware cost as in the case of FDMA or require complex operations and stringent power management as in the case of CDMA, which are not suitable for low-power M2M devices. For a detailed survey of M2M hybrid MAC protocols, one may refer to the study [4].

A common feature shared by the aforementioned hybrids is random-access. This feature makes them susceptible to collisions, which are amplified in densely populated networks even for small traffic loads. The susceptibility to collisions renders the protocols inefficient in M2M networks. Moreover, the delay sensitivity of data traffic is not considered, which can have adverse effects on the performance of the network. Therefore, in this paper, to address both the issues of contention collisions and data loss, we propose a hybrid medium access protocol named **or**thogonal coded **m**edium **a**ccess **c**ontrol (OrMAC). OrMAC extends the principle of distributed queuing collision avoidance protocol (DQCA) [8] to delay-sensitive M2M networks. The main contributions of the paper are as follows.

1. Use of pre-assigned orthogonal codes for channel contention to eliminate contention collisions.
2. Transmission prioritization based on the delay sensitivity of the data packet to mitigate the probability of losing delay-sensitive data.
3. The near-optimum throughput performance of DQCA is attributed to the use of two distributed logical queues. However, even with a single centralized logical queue—data transmission queue (DTQ)—we show that OrMAC can achieve better throughput performance than DQCA.

The preliminary part of this work was presented at a conference [9]. This paper is an extended version of [9] with enhancements made to the prioritization mechanism of the protocol (contribution no. 2). In particular, OrMAC determines the priority based on the urgency of the packets waiting for transmission in the DTQ to begin transmission in order to avoid data loss owing to expiration. New results include the throughput and discarded packet ratio; furthermore, additional results related to the packet delivery ratio and latency are also included. This paper is organized as follows. In Section 2, a detailed description of OrMAC is presented. Section 3 provides the simulation results and a discussion on the performance of OrMAC as compared to DQCA. Finally, Section 4 concludes this study.

## 2. OrMAC

OrMAC, just as DQCA, is a hybrid MAC protocol that can behave as a random-access-based contention scheme under low traffic environments and switch to a contention-free scheme under high traffic environments. However, a few features distinguish OrMAC from DQCA and we elaborate them in the following paragraphs alongside the description of OrMAC.

First, in OrMAC, the access point (AP) pre-assigns each node with a unique orthogonal code, which can be used for channel access request, whereas DQCA sets aside *n* contention slots that the nodes have to select and contend for channel access. The selection process of the contention slots in DQCA is random, which leads to contention collisions since the nodes are not aware that a particular slot has already been selected. Therefore, assigning each node entering the network with a unique orthogonal code, which is generated by a Walsh–Hadamard matrix [10], eliminates contention collisions, thereby removing the need of a contention resolution algorithm. For the sake of simplicity, we interchangeably refer to the orthogonal code used for channel access as the *contention signal*.

**Figure 1:**
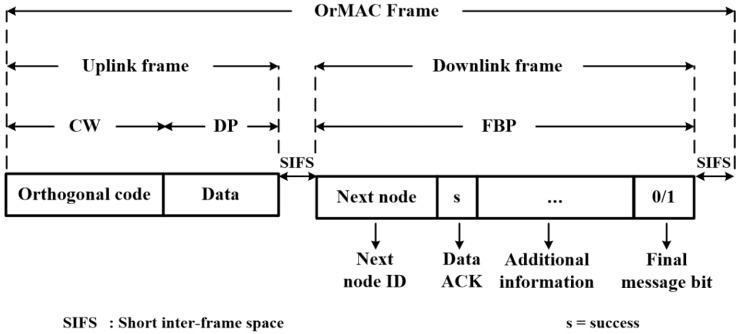
Structure of a single **or**thogonal coded **m**edium **a**ccess **c**ontrol (OrMAC) frame.

The frame structure of OrMAC, which is a modified version of DQCA, is shown in Figure 1.

A single frame of OrMAC consists of a *contention window* (CW), *data part* (DP), and quadripartite *feedback packet* (FBP). The quadripartite FBP further consists of the fields *Next node ID*, *Data ACK*, *Additional information*, and *Final message bit*.

(1) *Contention window*: In the CW, the data-ready nodes transmit their contention signal to gain channel access.
(2) *Data part*: The DP carries the data packet of the node that contended in one of the previous frames and obtains the channel access in the current frame. The size of DP is considered to be fixed and therefore, in cases where the size of the message is larger than that of the DP, the message is fragmented into sizes equal to the size of the DP. In order to indicate the final fragment of a fragmented message, the nodes include a ‘*final message bit*’ (not shown in the figure) in their data packet [11].
  The combination of CW and DP forms the uplink frame.
(3) *Feedback Packet*: The FBP is broadcasted by the AP, essentially forming the entire downlink frame, and it consists of the following fields: *Next node ID*, *Data ACK*, *Additional information*, and *Final message bit*. The *Next node ID* field carries the orthogonal code of the node that is given permission to transmit in the following frame. If the uplink data transmission in the current frame is not completed, the *Next node ID* remains the same and will continue to carry the code of the current transmitting node. The *Data ACK* field indicates the successful reception of the data packet in the current uplink frame. Similar to the *final message bit* of the DP (note the lowercase *f*), the *Final message bit* (note the use of uppercase *F*) field indicates the completed transmission of a fragmented message. The *Additional information* field can be used to carry miscellaneous information.

Short inter-frame spaces are inserted between the uplink and downlink frames to counterbalance the delays generated by the distance between the nodes and the AP by the turnaround times (switch between receive and transmit modes) and other internal processes.

**Figure 2:**
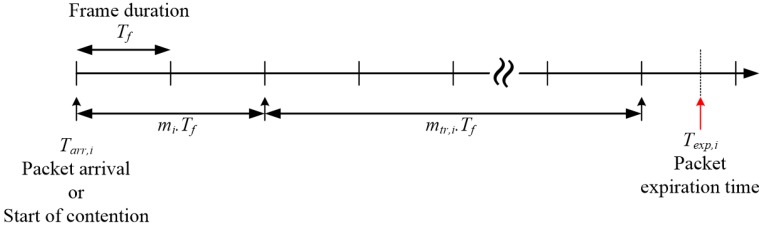
Timeline of the i*-*th data-ready node requesting channel access and waiting for its turn to transmit.

The second distinct feature of OrMAC is to prioritize the nodes for transmission based on the amount of delay or latency the data of a node can tolerate—i.e., delay sensitivity—unlike DQCA, which implements first-in first-out (FIFO). FIFO observes only the arrival times and may adversely affect the performance of a network with delay-sensitive traffic. Therefore, to support delay-sensitive networks, OrMAC allows data-ready nodes to determine their priority level depending on the delay sensitivity of their data and reflect the priority level information in the transmission power of the contention signal such that the contention signal arrives at the AP with received power of

(1) $$P_{i}^{R}\;=\;P_{max}\;-\;m_{i}\Delta P,\;i\in 1,\;2,\;3\ldots M$$

where $P_{max}$ is the maximum achievable received power, $m_{i}$ is the maximum number of frames that the i*-*th node can tolerate after which it should begin data transmission, M is the number of nodes, and ΔP is a non-zero constant ($\Delta P\ll \;P_{max}$). $P_{max}$ and ΔP are system parameters and are therefore known to the nodes and the AP. The value of ΔP has to be chosen such that $P_{i}^{R}$ is maintained above a certain SNR (signal-to-noise ratio) threshold. A higher $P^{R}$ indicates a smaller m, which further indicates a higher priority. Figure 2 illustrates the composition of the frames for the i*-*th data-ready node on a timeline.

Let us suppose that the i*-th* packet arrives at $T_{arr,i}$ and its transmission should be completed by $T_{exp,i}$, which depends on the traffic type. Considering the packet size, the number of frames to be occupied by the i*-th* packet is given by

(2) $$m_{tr,i}\;=\;\frac{l_{d,i}}{L_{D}}$$

where $l_{d,i}$ and $L_{D}$ are the size of the i*-th* packet and the size of *DP*, respectively, and [k] denotes the largest integer not greater than k. By subtracting, in reverse, the number of frames the packet will occupy ($m_{tr,i}$) from the total number of frames until packet expiration time ($T_{exp,i}$), a node can obtain the number of tolerable frames ($m_{i}$) until transmission begins. Then, in accordance with Equation (1), a node determines the transmission power of the contention signal. When the AP receives the contention signal, it extracts $m_{i}$ from $P_{i}^{R}$ and sets the priority according to the value of $m_{i}$. The node is subsequently placed in the DTQ according to $m_{i}$. DTQ is a logical queue that stores the nodes for transmission and is managed and monitored by the AP.

In order to monitor the expiration time, $T_{exp,i}$, the AP updates $m_{i}$ by one decrement after every frame; thus, the priority of the i*-*th waiting node is increased. In case the i*-th* node fails to obtain a channel when the value of $m_{i}$ becomes 0 (the minimum), the data is discarded. In case more than one node has the minimum m, the node whose arrival time is the earliest obtains the channel. If the arrival times are also the same, the AP randomly selects the winner. The nodes that fail to obtain the channel until m becomes 0 discard their data. We describe, with an example, the working of the priority assignment in the subsequent paragraph.

**Figure 3:**
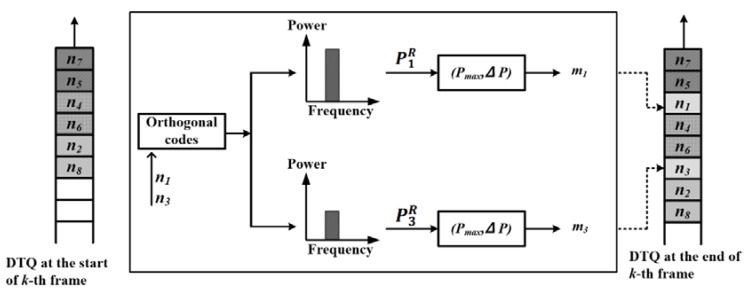
Example of the priority assignment operation in OrMAC.

Let us consider the *k-*th frame where nodes $n_{7}$, $n_{5}$, $n_{4}$, $n_{6}$, $n_{2}$, and $n_{8}$ are already queued in the ascending order of m in the DTQ, waiting for their turn to transmit. At the start of this frame, nodes $n_{1}$ and $n_{3}$, which have data to send, are contending for channel access (shown in Figure 3). On receiving the requests of nodes $n_{1}$ and $n_{3}$, the AP retrieves $P_{1}^{R}$ and $P_{3}^{R}$ and calculates $m_{1}$ and $m_{3}$, respectively. This step can be represented as a function $m_{i}\;=\;f\left(P_{i}^{R},P_{max},\;\Delta P\;\right)$. However, since $P_{max}$ and ΔP are constants, the function can simply be represented as $m_{i}\;=\;f\left(P_{i}^{R}\;\right)$. In this example, let us assume that $m_{1}$ falls between $m_{5}<\;m_{1}<\;m_{4}$ and that $m_{3}$ falls between $m_{6}<\;m_{3}<\;m_{2}$. Subsequently, the AP updates the DTQ in the following order $n_{7}$,$\;n_{5}$, $n_{1}$, $n_{4}$, $n_{6}$, $n_{3}$, $n_{2}$, and $n_{8}$.

### Operation Example of OrMAC

**Figure 4:**
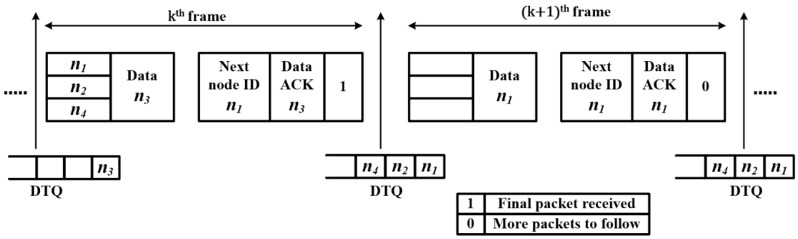
OrMAC operation between two consecutive frames.

The detailed operation of OrMAC is described in this section. Consider two consecutive frames, denoted by *k* and *k + 1*, as shown in Figure 4. In the *k-th* uplink frame, $n_{3}$ is transmitting its final data packet and $n_{1},n_{2},$ and $n_{4}$ are transmitting their contention signals. On receiving the contention signals of $n_{1},n_{2},$ and $n_{4}$, the AP obtains the respective $P^{R}$s and performs the extraction of m. Let us consider $m_{1}<\;m_{2}<\;m_{4}$. In the *k-th* downlink frame, since $n_{3}$ has completed its message transmission, the AP sets the *Next node ID* field to $n_{1}$ and the *Final message bit* field to 1, while $n_{2}$ and $n_{4}$ continue to wait in the queue. Simultaneously, the AP acknowledges the successful reception of the data packet (in this case, the final data packet of $n_{3}$) through the *Data ACK* field. In the *(k + 1)-th* uplink frame, no nodes are assumed to be contending for channel access; therefore, the CW field is empty while $n_{1}$ is transmitting its data packet. Assuming that $n_{1}$ did not complete transmitting its message in the *(k + 1)-th* frame, the AP sets the *Final message bit* field of the *(k + 1)-th* downlink frame to 0. Concurrently, the AP also updates the value of $m_{2}$ and $m_{4}$. Further, $n_{1}$ continues to transmit its data in the subsequent frame(s).

## 3. Numerical Experiments

### 3.1. Simulation Environment and System Parameters

Using MATLAB software, we developed an event-driven network simulator. We consider a star network topology where M nodes are randomly placed around the AP. The nodes generate data packets following a Poisson distribution with an average generation rate (λ) of one packet per second, unless specified otherwise. Each packet size equals 10 $L_{D}$ [8]. For the sake of simplicity, we considered three priority groups based on the permissible transmission delay shown in Table 1 [9,12], which also represents the delay sensitivity or the expiration time. Table 2 lists the system parameters.

### 3.2. Performance Metrics

#### 3.2.1. Packet Delivery Ratio (PDR),ρ

If $N_{g}$ is the number of data packets generated and $N_{r}$ is the number of data packets received successfully, the packet delivery ratio (PDR) can be defined as

(3) $$\rho ={(N_{r}/N}_{g})\times 100.$$

A data packet is considered to be discarded (a) in the case of DQCA, if the data packet arrives at the AP after the permissible transmission delay listed in Table 1; (b) in the case of OrMAC, if the data packet fails to be transmitted until the permissible transmission delay is expired, i.e., the case where m turned 0 before obtaining a channel. We do not consider any transmission error caused by the physical channel. Subsequently, the discarded packet ratio (DPR) is given by

(4) ζ=1−ρ.

#### 3.2.2. Latency, τ

If the j*-th* data packet was generated at a certain time $T_{g,j}$ and arrives at the destination at a certain time $T_{a,j}$, τ can be defined as the average time taken by the data packets to reach the AP and is given by

(5) $$\tau =\sum_{j=1}^{N}(T_{a,j}-T_{g,j})/N$$

where N is the total number of data packets received by the AP.

#### 3.2.3. Throughput, γ

If T denotes the duration for which the network was active, the throughput can be defined as the amount of data successfully delivered over T and is given by

(6) $$\gamma =L_{d}N_{r}/T.$$

### 3.3. Simulation Results

**Figure 5:**
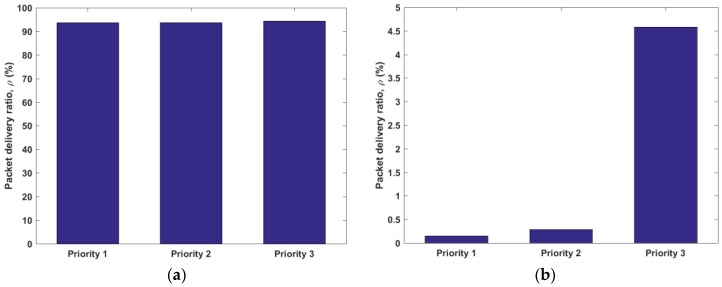
Packet delivery ratio (PDR) for each protocol with λ = 1.25 packet per second, (**a**) OrMAC (**b**) distributed queuing collision avoidance protocol (DQCA).

Using the aforementioned simulation parameters and performance metrics, we evaluated and compared the performances of DQCA and OrMAC protocols. Each simulation was carried out for a total of 1 × 10^6^ frames. Figure 5 shows the PDR achieved by the protocols for each priority group. With λ = 1.25, the figure shows that OrMAC can achieve very high PDR (and low DPR) across all three priority groups. The inherent feature of OrMAC to provide the opportunity to occupy a channel based on the delay sensitivity effectively improves the likelihood that the delay-sensitive packets obtain the channel earlier than others. The large room for delay in delay-tolerant packets, such as Priority 3, can be transferred to delay-sensitive packets, as it does not compromise the successful delivery of delay-tolerant packets while minimizing the loss of delay-sensitive packets. Moreover, since OrMAC updates the priority after every frame, the packet that enters the queue with a lower priority obtains a higher priority as the waiting time passes by and is eventually successfully transmitted. On the contrary, in DQCA, since the data packets are scheduled based on the FIFO principle without consideration of delay sensitivity, more delay-sensitive packets are lost. This behavior results in the low PDR performance of DQCA, which also suggests a very high DPR. Table 3 lists the mean PDR of the three priority groups versus λ by DQCA and OrMAC.

**Figure 6:**
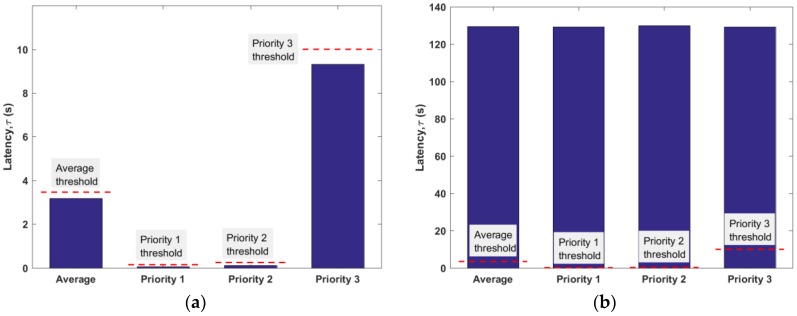
Latency for each protocol with λ = 1.25 packet per second, (**a**) OrMAC (**b**) DQCA. The dashed lines represent the permissible transmission delay given in Table 1.

Figure 6 outlines the latency experienced by each priority group and their average. On examining the latency of each priority group in DQCA, we observe that they obtain similar latencies, which are significantly above the permissible transmission delays. However, in OrMAC, each priority group obtains different latencies (in an increasing manner), which are sufficiently below the permissible transmission delays. This huge improvement can be attributed to the inherent feature of OrMAC to update the permissible transmission delay after every frame, thereby allocating the channel to a delay-sensitive packet at the cost of increasing the latency of the delay-tolerable packets. For instance, the latency of Priority 3 in OrMAC is very high compared to that of Priorities 1 and 2. Nonetheless, since the latency is below the permissible transmission delay, it does not have any detrimental effect.

**Figure 7:**
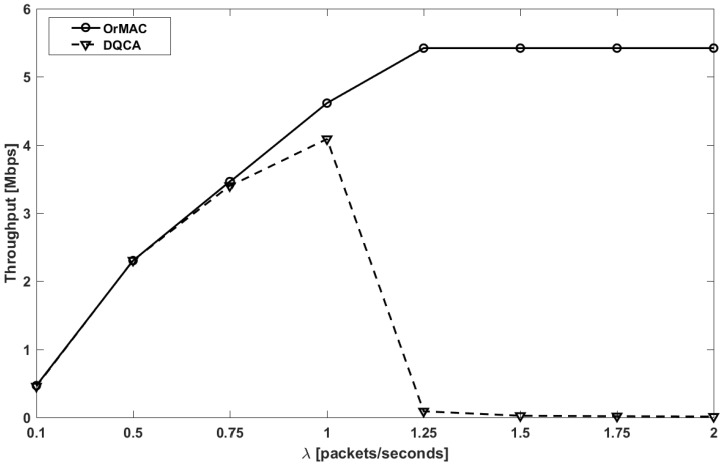
Throughput versus offered traffic load.

Figure 7 presents the evolution of throughput with respect to the offered traffic load. For smaller values of λ, both the schemes show a steady linear increment in the throughput. This trend in the throughput is attributed to the fact that the traffic load is sufficiently small for all the generated packets to be transmitted and eventually received successfully. After the steady increment, for higher values of λ (>1.25), the throughput of OrMAC begins to saturate toward a constant value. The saturation points represent the maximum achievable throughput. On the contrary, the throughput of DQCA degrades significantly after λ > 1. This detrimental effect can be attributed to the method of scheduling the transmissions in DQCA, i.e., FIFO, which does not consider the delay sensitivity. Such a method becomes a bottleneck when the rate at which the data packets leave the queue is significantly slower than the rate at which the data packets are generated. Consequently, the number of data packets in the queue increases, which inadvertently triggers a chain of events, i.e., an increase in the waiting time of the data packets in the queue, which leads to data packets exceeding their expiration time, and eventually the reduction in the number of data packets received successfully. In addition to FIFO, DQCA lacks the ability to discard expiry-bound data packets, which is reflected in the poor throughput performance. Therefore, for cases when λ > 1, the PDR of DQCA drastically reduces (see Table 3); consequently, the throughput deteriorates. From these analyses, it can be established that DQCA cannot handle higher rates of data generation for networks with delay-sensitive data unlike OrMAC. Note that for OrMAC, the PDR does not have any effect on the throughput since all the packets transmitted are received successfully, which is not the case with DQCA.

## 4. Conclusions

In this paper, a hybrid medium access protocol named OrMAC was proposed for delay-sensitive M2M networks. OrMAC aims to mitigate contention collisions by pre-assigning orthogonal codes, which serve as the channel contention signals, to the nodes entering the network. Furthermore, to avoid data loss owing to expiration, OrMAC prioritizes the data packets based on the urgency of the data packets, which is a key requirement for M2M networks with delay-sensitive data traffic. Therefore, OrMAC allows the nodes to control the transmission power of the contention signal in accordance with the delay sensitivity of the data.

Quantitative analysis through computer simulations shows that OrMAC outperforms DQCA in terms of the PDR, DPR, and latency. Further analysis shows that, for low traffic loads, DQCA and OrMAC achieve similar throughput. For higher traffic loads, it was observed that OrMAC can achieve the maximum achievable throughput, whereas DQCA suffers. Overall, we have demonstrated that OrMAC outperforms DQCA and it establishes the suitability of OrMAC for delay-sensitive M2M networks simply by employing orthogonal codes for channel access and prioritizing the nodes based on the delay sensitivity of a data.

## Figures and Tables

**Table 1:** Delay for each priority group (© 2016 IEEE).

Traffic Class	Delay	Priority
Voice and VOIP	<150 ms	1
Gaming and Two-way telemetry	<250 ms	2
Audio and Video streaming	<10 s	3

**Table 2:** System parameters (© 2016 IEEE).

Parameters	Value
*Common*	
Number of APs	1
Number of nodes, M	25
Length of DP, $L_{D}$	2312 bytes
Data rate	5.5 Mbps
FBP length	13 bytes
*Only for DQCA*	
ARS duration	10 μs
Number of control slots	3

**Table 3:** Mean PDR (%) for different values of λ.

λ	0.1	0.5	0.75	1	1.25	1.5	1.75	2
**DQCA**	99.99	99.78	98.19	88.29	1.67	0.45	0.33	0.24
**OrMAC**	100	100	100	100	93.93	78.29	67.11	58.79
